# A study on the value of narrow-band imaging (NBI) for the general investigation of a high-risk population of nasopharyngeal carcinoma (NPC)

**DOI:** 10.1186/s12957-018-1423-5

**Published:** 2018-07-04

**Authors:** Yong-Feng Si, Zhuo-Xia Deng, Jing-Jin Weng, Jin-Yuan Si, Gui-Ping Lan, Ben-Jian Zhang, Yong Yang, Bo Huang, Xing Han, Ying Qin, Yang-Da Qin, Wei-Ming Xiong, Bing Li

**Affiliations:** Department of Otolaryngology-Head and Neck Oncology, The People’s Hospital of Guangxi Zhuang Autonomous Region, No. 6 of Taoyuan Street, Nanning, 530021 Qingxiu District China

**Keywords:** Nasopharyngeal carcinoma, Census, Narrow-band imaging, Endoscopy

## Abstract

**Background:**

This study aims to explore the feasibility of narrow-band imaging (NBI) applied for the diagnostic screening of a high-risk population of nasopharyngeal carcinoma (NPC) and increase the accuracy rate of nasopharyngeal biopsy and the diagnosis rate of early-stage patients.

**Methods:**

The positive high-risk population of NPC to EB virus antibody was followed up. At the same time, serological screening and pharyngorhinoscopy were carried out. The specific methods were as follows: (1) all subjects received nasopharyngeal examinations through both the HD endoscopic white light mode (WL) and NBI mode, (2) nasopharyngeal biopsy was conducted on positive subjects with microscopic examination, and, finally, (3) a comparative analysis was conducted between the biopsy pathology results and microscopy results. In addition, the following comparative indicators were recorded under different modes: sensitivity, specificity, accuracy, positive likelihood ratio, and negative likelihood ratio. Then, the area under the ROC curve and the kappa coefficient were calculated.

**Results:**

A total of 115 subjects were detected to be positive by microscopic examination under the WL mode. Among these subjects, 19 subjects were diagnosed with NPC. In addition, 24 subjects were detected to be positive by microscopic examination under the NBI mode. Among these subjects, 23 subjects were diagnosed with NPC. Under the WL mode, the specific values of the comparative indicators were as follows: sensitivity, 82.61%; specificity, 0%; and area under the ROC curve, 0.413. Furthermore, the WL mode in the diagnosis on the high-risk population of NPC exhibited poor consistency with the biopsy pathology results (kappa coefficient = − 0.069). Under the NBI mode, the specific values of the comparative indicators were as follows: sensitivity, 100%; specificity, 98.96%; and area under the ROC curve, 0.995. Furthermore, the NBI mode in the diagnosis on the high-risk population of NPC exhibited relatively satisfactory consistency with the biopsy pathology results (kappa coefficient = 0.973). Therefore, the NBI mode is significantly superior to the WL mode.

**Conclusion:**

NBI endoscopic examinations should be conducted on a routine basis for high-risk populations of NPC. This can decrease the frequency of biopsies and enhance diagnostic effects.

## Background

Nasopharyngeal carcinoma (NPC) is a common malignant head and neck tumor [1]. It has been verified by studies that the 5-year survival rate of NPC can reach up to 90% [2], and the main reason for treatment failure of advanced NPC lies in the distant metastasis [3, 4]. Hence, the most effective approach to enhance the long-term therapeutic effect of NPC is early diagnosis and treatment, and early diagnosis is the necessary condition to early treatment. Through testing for peripheral blood Epstein-Barr (EB) virus antibodies, the high-risk population of NPC could be screened out and undergo nasopharyngeal examinations. For subjects with abnormities, biopsies were conducted. When subjects were pathologically diagnosed with NPC, they are treated accordingly. General NPC investigation covers two key points: (1) in serological screening, the high-risk population of NPC was screened out via the EB virus antibody test [5], and (2) in diagnosis screening, the high-risk population of NPC underwent nasopharyngeal examinations [6]. During the screening, biopsy rate was higher, while positive pathology rate was lower. This was because the anatomical position of the nasopharyngeal region is relatively deep, nasopharyngeal mucosal inflammation tends to occur, and the mucosal appearance is not smooth. Part of these NPCs was of submucosal type. Furthermore, standard endoscopic examination revealed that the mucosal lesions were not clear, and no biopsy position was identified, which lead a lower diagnosis rate of the biopsy. At present, the WL mode is commonly used in endoscopic examinations. In white light mode (WL), nasopharyngeal masses are considered positive [7]. However, no subtle changes in nasopharyngeal mucosal blood vessels are likely to be observed, which has adverse effects on diagnostic screening results. Narrow-band imaging (NBI) can display these abnormal subtle submucosal vessels [8]. The novel endoscopic findings in nasopharyngeal carcinoma (NPC) under narrow-band imaging (NBI) were as followed: brownish spots, irregular microvascular pattern (IMVP), light crests, and side-difference. Hence, it can increase the diagnostic rate of subtle lesions or superficial lesions. Since 2011, our department has applied NBI for NPC diagnosis and has presented satisfactory effects [9]. Moreover, other scholars have carried out studies on head and neck carcinomas and verified that NBI outmatches common approaches [10–12]. However, studies on NBI focused on outpatients or inpatients [13], and the majority of the existing general investigations are mainly applicable to indirect pharyngorhinoscopy or hard-tube pharyngorhinoscopy. In addition, literature on general NPC investigations have rarely reported on NBI. Therefore, this technology was applied to the diagnostic screening of a high-risk population of NPC, in order to explore the role of NBI in general investigations. The results are reported below.

## Methods

### Clinical materials

This was a study, in which a general NPC investigation was carried out by the People’s Hospital of Guangxi Zhuang Autonomous Region in collaboration with the Guangxi Cangwu Nasopharyngeal Carcinoma Prevention Institute, Bobai People’s Hospital and Luchuan People’s Hospital from October 2013 to December 2014. All the eligible subjects were asked to participate in the screening tests. Inclusion criteria were as follows: (1) age between 29 and 60 years, (2) without prevalent NPC, and (3) having a good physical and psychological self-consciousness. Those with history of nasopharyngeal carcinoma were excluded. The high-risk population of NPC was selected by conducting EB virus antibody tests of the peripheral venous blood collected from the subjects. Starting August 2015, the high-risk population of NPC was followed up, and examinations included serological test of EB virus antibody and nasopharyngeal endoscopy. Among the 5960 persons who are at high risk in these three counties, three were with history of nasopharyngeal carcinoma, 39 persons were with EB virus antibody (+) turned negative, and finally 5918 were enrolled into the study. Informed consent was obtained.

### Observation indicators and test methods

#### Nasopharyngeal endoscopy and result assessment

An HD electronic nasopharyngolarygnoscope with both WL and NBI modes (VISERA Pro OTV-S7Pro, Olympus, Japan) was adopted. In this study, all subjects received nasopharyngeal examinations through the HD electronic nasopharyngolarygnoscope. Examination steps: a mixed solution of 1% tetracaine + 1% ephedrine was used for contraction, and the nasal cavity and nasal nasopharyngeal mucosa were sterilized twice. Through the nasal cavity, the HD electronic nasopharyngolarygnoscope was placed in the nasopharyngeal region for the examination. The examinations were first carried out under WL mode, and subsequently under NBI mode; and the examination results were recorded. The result judgment standards were as follows:

1. Examinations under the WL mode: positive symptoms include mass lesion, apophysis and rough superficial mucosa, anabrosis, and local mucosa color abnormity in the nasopharyngeal region. The remaining symptoms were regarded as negative.
2. Examinations under the NBI mode: (1) abnormal and positive symptoms include the increased superficial vessels of the mucosa, increased (expanded) vessel diameter, disordered texture, local circuitry, intensively distributed punctiform vessels, and lumbriciform, or rope-strip-shaped vascular disruptions [5]. (2) Negative symptoms were as follows: the texture and level of lymphoid tissues can be clearly distinguished, and no superficial blood vessels with morphologic abnormality could be observed in the mucosal surfaces.

#### Indications of nasopharyngeal biopsy and tumor staging method

Biopsy indications: endoscopic examination was conducted regularly for positive subjects under either WL mode or NBI mode, and negative subjects were examined both under WL mode and NBI mode. Nasopharyngeal tissue biopsy pathology results were regarded as the diagnostic criterion of NPC. The pathological diagnosis was conducted with reference to the World Health Organization (WHO) NPC histopathological typing [14]. For patients confirmed with NPC, the following examinations are carried out: nasopharyngeal skull base and cervical region MRIs, chest X-ray, abdominal ultrasound, and bone ECT scanning. Finally, the tumors were classified by stage according to the AJCC (Version 7) NPC staging scheme [15].

### Statistical analysis

Statistical software SPSS 17.0(Chicago, IL, USA) was adopted, and chi-squared test was used to compare the corresponding rates. A *P* value < 0.05 was considered statistically significant.

## Results

### Follow-up conditions

Up to December 30, 2015, a total of 5918 high-risk subjects (male, 2850; female, 3068) were followed up. The mean age of these subjects was 48. Among them, 119 were positive patients, as detected by endoscopic examination, with the rate of 2.01%.

### Microscopic examination results of the two modes

Among the 5918 subjects that were followed up, the WL mode microscopic examination revealed 115 positive cases and 5803 negative cases, while the NBI mode microscopic examination revealed 24 positive cases (0.41%) and 5894 negative cases. The difference among these positive rates for both modes was statistically significant (*P* < 0.001, Table 1).

**Table 1:** Two modes of microscopic examination results

	White light mode	
NBI mode	Positive	Negative
Positive	20	4
Negative	95	5799

### Nasopharyngeal biopsy condition and microscopic examination result comparison of both modes

Among all subjects, 119 cases were positive by nasopharyngeal microscopy, in which 23 cases were diagnosed with NPC and 96 cases were pathologically diagnosed with chronic inflammation of the nasopharyngeal mucosa.

#### Corresponding correlation between microscopy results under both modes and biopsy pathology results

Among the 119 cases who underwent nasopharyngeal biopsy, 24 cases were positive patients by NBI microscopy. Among these 24 cases, 23 cases were diagnosed with NPC (95.83%) and one case was diagnosed with chronic inflammation of the nasopharyngeal mucosa. Under the WL mode, 115 positive cases received nasopharyngeal biopsy, in which 19 cases were diagnosed with NPC (Table 2).

**Table 2:** The relationship between the two microscopic examination results and biopsy results

	Pathological examination (*n*)		Result (%)			
	+	–	Sensitivity	Specificity	Predictive value	
					Positive	Negative
White light						
+	19	96	82.61	0.00	0.17	0
−	4	0				
NBI						
+	23	1	100.00	98.96	0.96	1
−	0	95				

#### The corresponding correlation between microscopy features under both modes and biopsy pathology results

Under the WL mode, NPC color abnormity rate was 73.91%, which was more than that of the chronic inflammation of the nasopharyngeal mucosa (57.29%), but the difference was not statistically significant (*P* = 0.143). Furthermore, morphologic abnormality (apophysis or anabrosis) rate was 82.61%, which was obviously higher than that of the chronic inflammation of the nasopharyngeal mucosa (60.42%), and the difference was statistically significant (*P* = 0.045). Under the NBI mode, the increase rate, expansion rate, and morphologic abnormality rate of blood vessels with NPC were all significantly more than those of the chronic inflammation of the nasopharyngeal mucosa (*P* < 0.001) (Table 3).

**Table 3:** The relationship between the two modes of microscopic examination and biopsy results

Endoscopic mode	Endoscopic features	Mucositis (*n* = 96)	Nasopharyngeal carcinoma (*n* = 23)	*P* value
White light	Color abnormal	55	17	0.143
	Shape abnormal	58	19	0.045
NBI	Vascular increased	1	15	0.000
	Vascular dilation	1	17	0.000
	Abnormal vascular morphology	0	19	0.000

#### Comparison of microscopic examination results under two modes

**Fig. 1:**
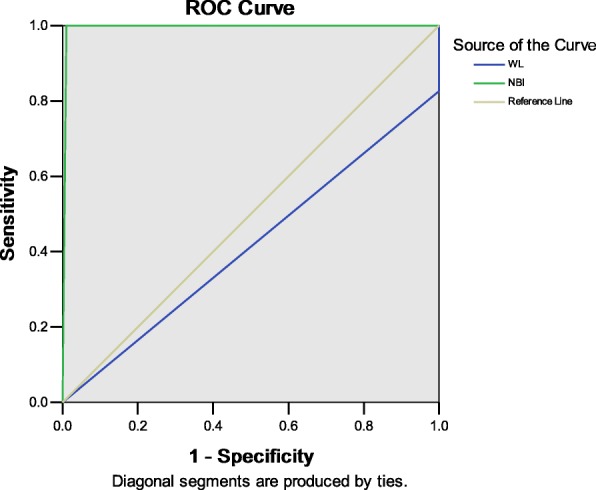
Comparison of ROC curve of two patterns for the diagnosis of nasopharyngeal carcinoma

Sensitivity, specificity, and accuracy under the NBI mode were superior to those under the WL mode (*P* < 0.05). However, under the NBI mode, the consistency between the diagnosis of the high-risk population of NPC and biopsy pathology results were very satisfactory (kappa coefficient = 0.973), while such consistency under the WL mode was poor (kappa coefficient) (Table 4 and Fig. 1).

**Table 4:** Comparison of the accuracy of two models in the diagnosis of nasopharyngeal carcinoma

Mode	Area	*P* value	95% CI	
			Lower limit	Upper limit
White light	0.413	0.196	0.272	0.554
NBI	0.995	0.000	0.983	1.007

### Staging results of both NPC groups

**Fig. 2:**
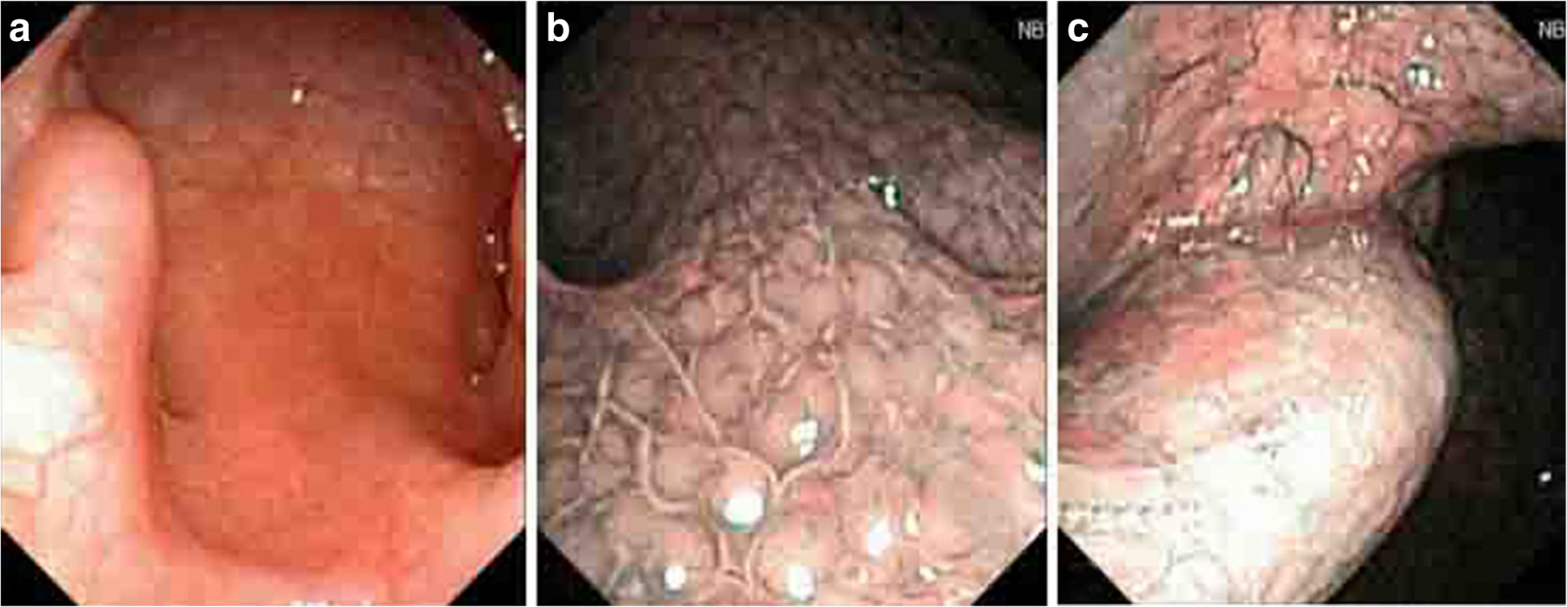
**a** Smooth nasopharyngeal mucosa was found under the WL mode, and each anatomical sign was clear. **b** The mucosa-associated lymphoid tissue under the NBI mode was even, and no abnormal proliferating superficial vessels were found. **c** No clustered vessel growth was observed in the local mucosa under the NBI mode

**Fig. 3:**
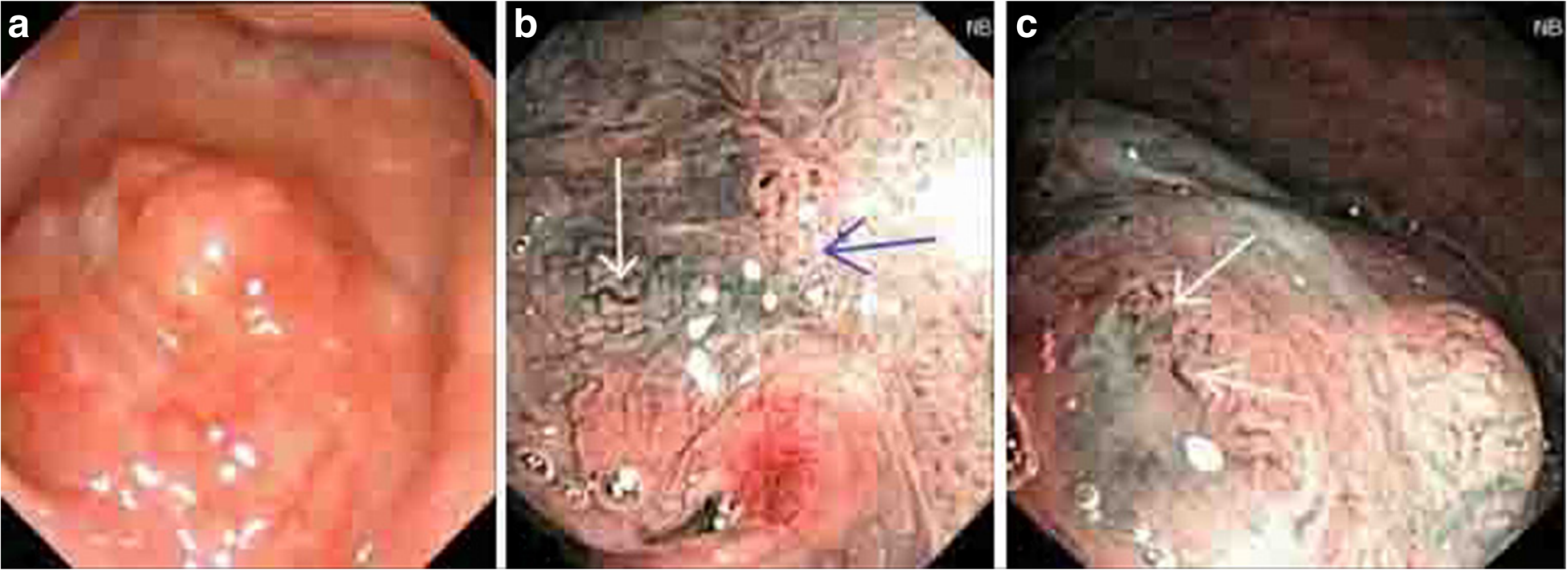
**a** The nodular neoplasm was observed in the nasopharyngeal with a rough superficial mucosa, which occupied a large area of the nasopharyngeal cavity. The pharyngeal recess was compressed. **b** The mucosal superficial vessel morphology was irregular. The white arrow shows the circuitous vessels, and the blue arrow shows the looped vessels. **c** The white arrow shows the distorted and locally expanded superficial vessels. Pathological diagnosis: undifferentiated and non-keratinizing carcinoma

**Fig. 4:**
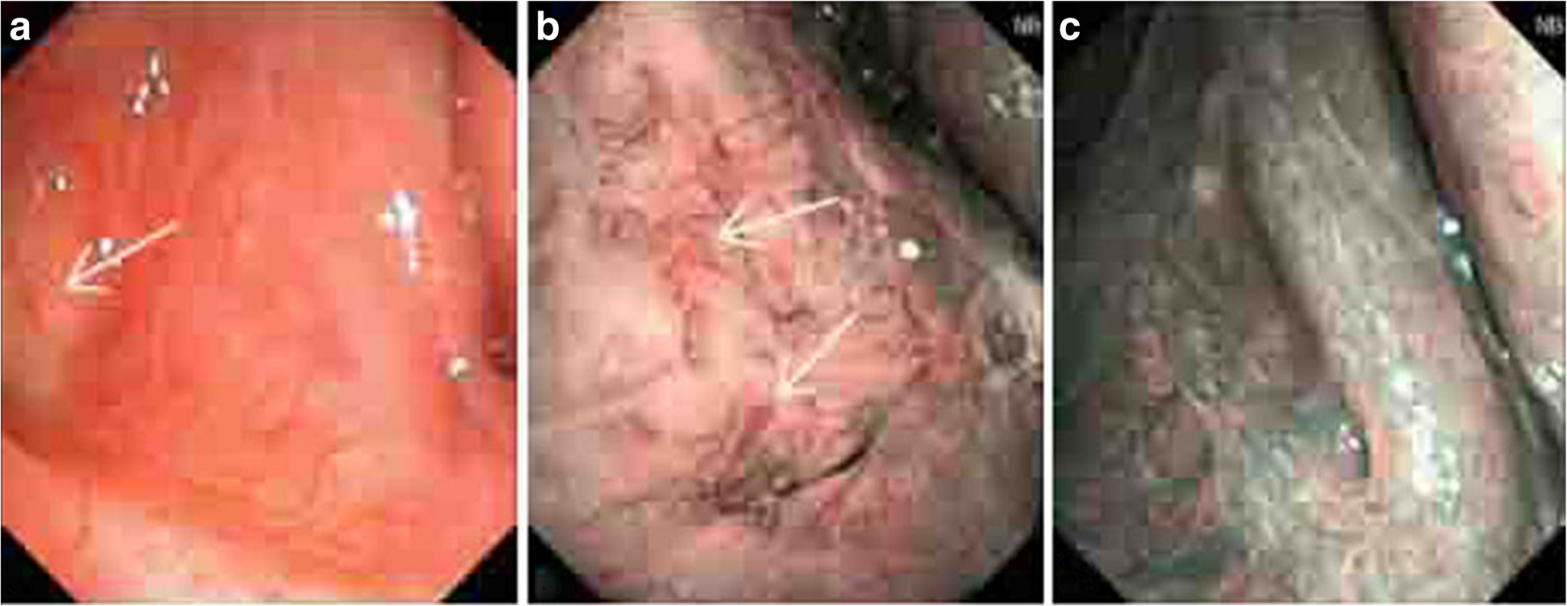
**a** The smooth nasopharyngeal mucosa was found under the WL mode, which had a ruddy color. No obvious tumor profile was found. **b** Within the range guided by the arrow in the WL view field under the NBI mode, a disordered distribution and the circuitous superficial vessels of the mucosa were found. The walking shape was parallel to the mucosa. **c** Right nasopharyngeal superficial vessels under the NBI mode were observed, with clear texture. No typical and special-shaped vessels were revealed. The nasopharyngeal tissue was submitted for pathological examination on the position guided by the arrow, with a definite diagnosis of undifferentiated and non-keratinizing carcinoma

**Fig. 5:**
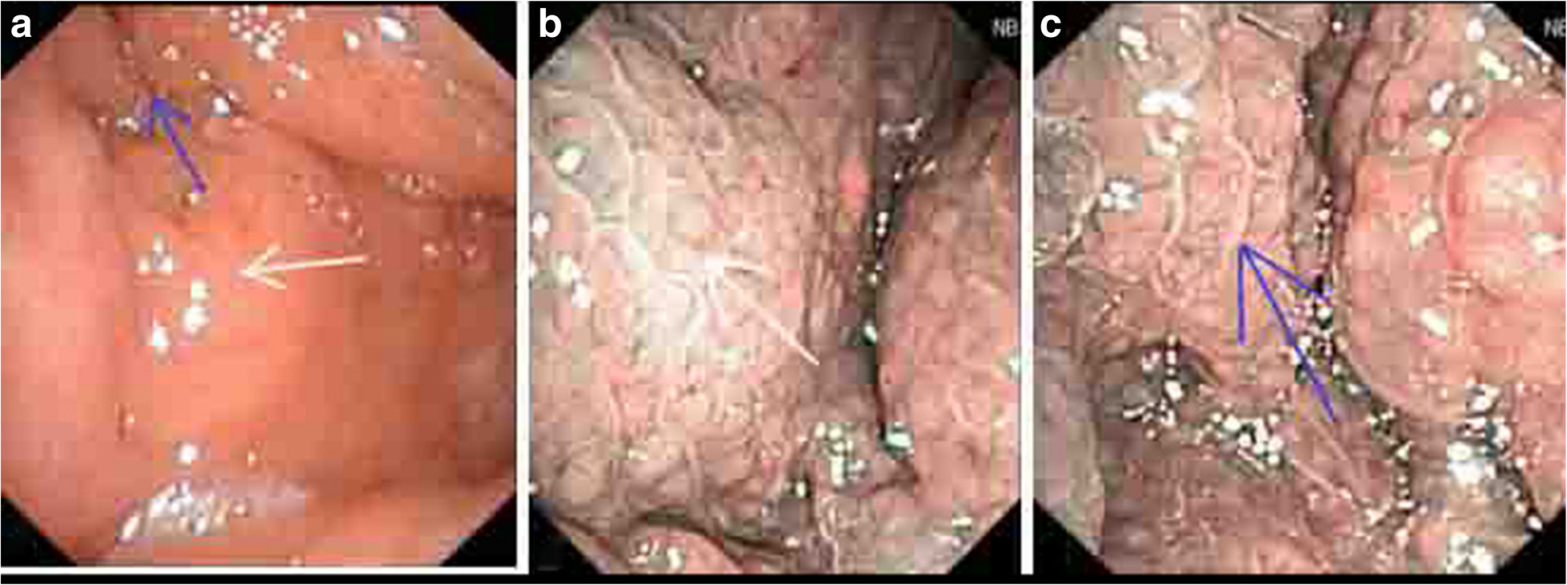
**a** The nasopharyngeal posterior wall was uplifted under the WL mode. The left pharyngeal recess was full. The nodular neoplasm was observed. **b** The posterior wall was uplifted due to the thickened mucosa. Proliferous lymphoid tissues were observed under the mucosa (white arrow). **c** The neoplasm parenchyma in the left pharyngeal recess refers to proliferous lymphoid tissues (blue arrow). The nasopharyngeal tissue was submitted for pathological examination on the position guided by the arrow, with a definite diagnosis of chronic inflammation of the nasopharyngeal mucosa

A total of 23 cases were diagnosed with NPC, and staging was carried out according to the AJCC (Version 7) NPC staging scheme. The results are as follows: T1, eight cases; T2, 13 cases; and T3, two cases. Moreover, all 23 cases had no lymphatic metastasis. The staging of NPC diagnosed under the NBI mode is shown below: stage I, eight cases; stage II, 13 cases; and stage III, two cases. The diagnosis rate of the early stage (stage I and II) was 91.3%. For the WL mode: stage I, four cases; stage II, 13 cases; and stage III, two cases. The diagnosis rate of the early stage (stage I and II) was 89.5% (Table 5, Figs. 2, 3, 4 and 5).

**Table 5:** Staging comparison of two groups of nasopharyngeal carcinoma

Mode	*n*	Stage 1	Stage 2	Stage 3	Stage 4
White light	19	4	13	2	0
NBI	23	8	13	2	0

## Discussion

Being endemic in South China, the five-year survival rate of early-stage NPC can reach up to 89.7% [16], while that of middle-late stage NPC decreased to 75% [17]. For the steps of the general NPC investigation, through EB virus serological screening, the high-risk population of NPC was identified, and cases with NPC were diagnostically screened out. In this study, a follow-up by microscopy was carried out according to the above steps, and biopsy was carried out for the population with abnormalities determined by microscopic examination. Previously, WL mode endoscopic systems such as the nasal endoscope, fiber, or electronic nasopharyngolarygnoscope were adopted to carry out the diagnostic screening of NPC. It is relatively difficult to display the subtle vessels of the nasopharyngeal mucosa by the WL mode endoscopic system, which led to failure in the recognition of subtle changes in vessels. This resulted to ① the misdiagnosis of the nasopharyngeal micro-carcinoma and ② the low diagnosis rate of nasopharyngeal biopsy due to difficulty in distinguishing nasopharyngeal inflammatory neoplasms from cancerous lesions. NBI can filter out the red light with the longest wave in the common white light through the narrowband light filter, and the green light and blue light of the narrowband spectrum can be reserved. The hemoglobin in submucosal vessels can absorb much more green light and blue light for the optical property. Thus, the contrast ratio between the mucosal epithelia and submucosal vessels would increase, which is conducive for identifying the morphologic changes of the tumor vessel.

In current literature, under the WL mode, 115 cases were diagnosed with abnormities, in which 19 cases were diagnosed with NPC, which was significantly more than that under the NBI mode (24 cases). This indicates that these 115 cases received nasopharyngeal biopsy under the WL mode. This was familiar with that of a previous study, where the nasopharyngeal endoscopy coupled with NBI was able to provide a rapid, convenient, and highly reliable screening for high-risk populations [18]. Few literature have reported the indications and standards of biopsy. Xu S et al. [19] believed that the diagnosis rate of biopsy under the nasal endoscope is higher than that through the oral cavity, but the specific indications of the biopsy were not mentioned. Different from microscopic examinations, a biopsy holds the advantage of identifying the diagnosis; while its disadvantages mainly include infection, hemorrhage and cancer cell detachment, and implantation metastasis due to the traumatic examination. Therefore, biopsy may lead to bleeding in patients with coagulopathy [20]. In the present data, color abnormity and morphologic abnormality were regarded as biopsy indications, and it was found that the rate of the two indications above in mucosal inflammation exceeded 50%. Hence, at present, there is an urgent need to seek for an effective method to guide the inclusion criteria for nasopharyngeal tissue biopsy.

Some research indicated that [21, 22] early-stage tumors tend to be accompanied with changes in mucosal superficial vascular structures such as the vascular morphology changes and morphology changes of new vessels. Similar to the above research results, in the present data, the increase rate, expansion rate, and morphologic abnormality rate of these blood vessels with NPC were all significantly more than those of the chronic inflammation of the nasopharyngeal mucosa. Based on such change in the early stage of tumors, during the application of NBI, mucosal vessels are revealed through blue and green light, and mucosal tissues and blood vessel morphologies are emphasized by means of optical imaging emphasis technology. This can improve the contrast performance of the images and increase the differential diagnosis rate. It has been reported by studies that the NBI endoscopic system was applied to the early diagnosis and differential diagnosis of other malignant tumors [16, 17]. Recently, it was reported that NPC diagnosis by NBI has been carried out in clinical and relevant fundamental studies [21–23]. All results verified the significance of NBI in NPC diagnosis. In previous studies, similar conditions were reached [9]. Under the NBI mode, the diagnosis accordance rates of cases with stage I and II NPC were 100.0% (5/5) and 85.7% (6/7), respectively, which were significantly higher than those under the WL mode (− 0 and 14.3% [1/7]), and the overall diagnosis accordance rate under the NBI mode was 93.0%. Subsequently, Madana et al. [23] verified by research that the NBI mode was superior to the WL mode, because NBI can display tumor vessels better. In the present data, vascular morphologic abnormality under the NBI mode reached up to 82.61%. Compared with the common WL mode endoscope, the endoscopic system with the NBI mode can freely switch over to the two modes once without placement of another endoscope. In this study, the NBI HD endoscope was applied for the general investigation, and the consistency with the biopsy pathology results was very satisfactory (kappa coefficient = 0.973). Moreover, sensitivity, specificity, and the area under the ROC curve were significantly superior to those under the WL mode; and the expenses were lower than that under the WL mode. Ni XG et al. [24] also obtained similar results, in which sensitivity, specificity, predicted positive value, and predicted negative value during the NBI endoscopy of the NPC were 80.6, 91.7, 96.7, and 61.1%, respectively. The present corresponding data were slightly higher than those above, and 2011–2012 levels were obtained (0.93). It is inferred that the above results are correlated with the increase in cases, which further accumulated and promoted experience. Different from other scholars, our group data was mainly applied to diagnostic screening and the number of examinations was greater. At present, few literature have reported that NBI technology can be applied to general NPC investigations. At present, due to the varied positive differences of the nasopharyngeal NBI mode [25], we consider that abnormal symptoms include disordered texture of the superficial vessels of the mucosa, local circuitry, intensively distributed punctiform vessels, and lumbriciform or rope-strip-shaped vascular disruption. In our group of patients, one positive patient was under NBI mode in the microscopic examination for research, with the pathology of nasopharyngeal mucosal chronic inflammation, and NBI feature was that the mucosal vessels were in local circuitry.

Since the nasopharyngeal position is relatively deep, early-stage NPC symptoms were obvious. In particular, it can be easily missed and diagnosis could be delayed for subjects with no lymphatic metastasis. For positive patients with the EB virus antibody, once a lymphatic metastasis is found, the patients can be early diagnosed in a timely manner, in general. At the earlier stage, we have also come to a similar conclusion through tracking and following up other areas [26]. In our group data, eight I-period NPC cases were found under NBI mode, and only four I-period NPC cases were found under WL mode. Thong et al. [27] also considered that the NBI mode was beneficial to the early stage of the tumor, because tumors in the early stage may have no obvious neoplasm uplifting.

## Conclusions

These present research results revealed that the sensitivity, specificity, and accuracy of the NBI mode are relatively higher than those of the WL mode for the diagnosis of NPC. Through the authenticity and reliability evaluation of these two modes, it was found that the NBI mode was superior to the WL mode. Therefore, we consider that endoscopic examination under the NBI mode should be applied to the diagnostic protocol of NPC, in order to significantly reduce the biopsy rate of non-NPC patients, relieve the subject’s pain, reduce the general investigation fund expenditure, and early detect NPC in patients.

## Abbreviations

EB: Epstein-Barr
NBI: Narrow-band imaging
NPC: Nasopharyngeal carcinoma
WHO: World Health Organization
WL: White light mode
